# Atom economy and green elimination of nitric oxide using ZrN powders

**DOI:** 10.1098/rsos.171516

**Published:** 2018-05-30

**Authors:** Ning Chen, Jigang Wang, Wenyan Yin, Zhen Li, Peishen Li, Ming Guo, Qiang Wang, Chunlei Li, Changzheng Wang, Shaowei Chen

**Affiliations:** 1 Laboratory for Micro-sized Functional Materials and College of Elementary Education and Department of Chemistry, Capital Normal University, Beijing, 100048, People's Republic of China; 2 Key Laboratory for Biomedical Effects of Nanomaterials and Nanosafety Institute of High Energy Physics, Chinese Academy of Sciences, Beijing, 100049, People's Republic of China; 3 Beijing Key Laboratory of Functional Materials for Building Structure and Environment Remediation, Beijing University of Civil Engineering and Architecture, Beijing, 100044, People's Republic of China; 4 Department of Chemistry and Biochemistry, University of California, 1156 High Street, Santa Cruz, CA 95064, USA

**Keywords:** green elimination, nitric oxide, zirconium nitride

## Abstract

Nitric oxide (NO) may cause serious environmental problems, such as acid rain, haze weather, global warming and even death. Herein, a new low-cost, highly efficient and green method for the elimination of NO using zirconium nitride (ZrN) is reported for the first time, which does not produce any waste or any by-product. Relevant experimental parameters, such as reaction temperature and gas concentration, were investigated to explore the reaction mechanism. Interestingly, NO can be easily decomposed into nitrogen (N_2_) by ZrN powders at 600°C with ZrN simultaneously transformed into zirconium dioxide (ZrO_2_) gradually. The time for the complete conversion of NO into N_2_ was approximately 14 h over 0.5 g of ZrN at a NO concentration of 500 ppm. This green elimination process of NO demonstrated good atom economy and practical significance in mitigating environmental problems.

## 1. Introduction

Emission of nitrogen oxides (NO*_x_*) produced from stationary and mobile combustion sources is one of the major contributors to atmospheric contamination [1]. In fact, NO*_x_* has been known to lead to worsening of local air quality, regional acid rain pollution as well as photochemical smog [2,3] and undesirable severe damage to human health [4]. With increasingly stringent NO*_x_* emission regulations, extensive efforts have been devoted to the development of new technologies for the elimination of NO*_x_* emission. Catalytic oxidation of nitric oxide (NO) plays a critical role in NO*_x_* storage and reduction [5,6]; and continuously regenerating trap [7] and selective catalytic reduction (SCR) [8–10] have been the main technologies for NO*_x_* removal. Among these, catalytic oxidation based on CuO*_x_*/Al_2_O_3_ and CuO*_x_*/LaO*_x_*/Al_2_O_3_ has been widely used in the treatment of automobile exhaust, which typically operates in the temperature range of 200–400°C [11], yet only less than 50% of NO can be eliminated. Note that for gasoline vehicles, the exhaust gas temperature may be up to 1000°C, which can lead to deactivation of the catalysts. SCR is of particular interest because it has been successfully employed for the reduction of NO*_x_* by NH_3_: 4NO + 4NH_3 _+ O_2_ = 4N_2_ + 6H_2_O. However, the use of ammonia increases the cost of operation and raises safety issues due to its corrosive nature. Furthermore, in the SCR reaction, NO and ammonia are introduced into the reaction vessel at a 1 : 1 molar ratio, and at insufficient reactants, there may be an outflow of excess ammonia, resulting in secondary pollution. Therefore, it is urgently needed to develop ‘green' and thermostable procedures for the treatment of gasoline vehicle exhaust especially for the removal of NO*_x_*.

Within this context, transition metal nitrides (e.g. TiN, GaN, etc.) have been attracting attention as viable candidates for NO*_x_* removal, although they are better known for improving the lifetime and performance of cutting tools [12,13]. Among these, zirconium nitride (ZrN) has been extensively studied owing to its excellent physicomechanical and tribological properties [14,15]. For instance, ZrN possesses high hardness, good electrical conductivity and low chemical reactivity [16], and has been used as wear resistant coatings on cutting tools [17], and as decorative films for scratch resistance and colouring in the jewellery industry [18]. Thin films of ZrN have also been used as diffusion barriers in integrated circuits [19]. However, up to now, reports have remained scarce involving the application of ZrN for the elimination of pollution gases. Currently, the synthetic methods of ZrN mainly include reactive magnetron sputtering, gas–solid reaction and physical vapour deposition-based methods [20,21], such as pulsed laser deposition [22,23], chemical beam epitaxy [24], ion plating [25] and vacuum evaporation [26]. Among these methods, gas–solid method is the most simple and economical.

In this study, ZrN powders were synthesized by gas–solid reactions of Zr powders under a N_2_ gas-flow and demonstrated apparent reactivity in the reduction of NO by the following reaction: 2ZrN + 4NO = 2ZrO_2_ + 3N_2_. In a typical experiment, 0.5 g of ZrN powder was sufficient for complete reduction of 500 ppm NO gas for up to 14 h at 600°C. The *in vitro* cytotoxicity evaluation and haemolysis assays have shown that the reaction product, zirconium dioxide (ZrO_2_), may be used as a biocompatible material for bone tissue engineering and regenerative medicine [27] and the other reaction product, N_2_, may be used as a protective gas or converted into liquid nitrogen for other purposes. In addition, no ammonia was used and there was no concern of corrosion for the reaction apparatus as well as secondary pollution. This suggests that the reaction process is atom economy and green chemistry in nature. Importantly, as NO was effectively removed at elevated temperatures, the results suggest that ZrN may be used as a green catalyst for gasoline vehicle exhaust treatment that runs at harsh high temperatures.

## 2. Experimental

### 2.1. Material preparation

All chemicals were purchased from Alfa Aesar and used as received without further purification. To prepare ZrN, zirconium powders were placed in a square boat and heated at 1200°C in a corundum tube under nitrogen for 12 h [28].

### 2.2. Material characterizations

The phase structures and morphologies of the ZrN powders prepared above were characterized by powder X-ray diffraction (XRD, Bruker D8 with Cu K*α* radiation, *λ* = 1.54 Å) and X-ray photoelectron spectroscopy (XPS, ESCALAB 250 with Al K*α* radiation) measurements. The lattice fringes of the obtained samples and the corresponding selected-area electron diffraction (SAED) patterns were examined by using a high-resolution transmission electron microscope (HRTEM, JEOL 2010F, 200 kV).

### 2.3. Elimination reduction of nitric oxide

Elimination reduction of NO was performed in a fixed-bed quartz tube reactor with an internal diameter of 6 mm [29]. The ZrN powders were ground to 40–60 mesh and placed on quartz wool held in the reactor, and the reactor was heated by a vertical electrical furnace. The total flow rate was 250 ml min^−1^ (room temperature), the mass of ZrN was 500 mg, and the corresponding gas hourly space velocity was 10 × 10^4^ cm^3^ g^−1^ h^−1^, which was evaluated by the following expression:

2.1 $$\text{GHSV}=\frac{q_{v}}{\pi hr^{2}},$$

where *q_v_* is the total flow rate, *h* is the height of the reactant in the reactor and *r* is the radius of the reactor [30]. The concentration of NO was monitored by an online gas chromatographic analyser equipped with a flame photometric detector (Beijing Beifen-Ruili 3420A). The NO conversion ratio was calculated by the following equation [31]:

2.2 $${\text{NO}}_{\mathrm{conversion}}=\frac{{\left[\text{NO}\right]}_{\mathrm{in}}-{\left[\text{NO}\right]}_{\mathrm{out}}}{{\left[\text{NO}\right]}_{\mathrm{in}}}\times 100\%,$$

where [NO]_in_ and [NO]_out_ refer to the NO concentration at the inlet and outlet, respectively.

## 3. Results and discussion

### 3.1. X-ray photoelectron spectroscopy characterization

**Figure 1.**
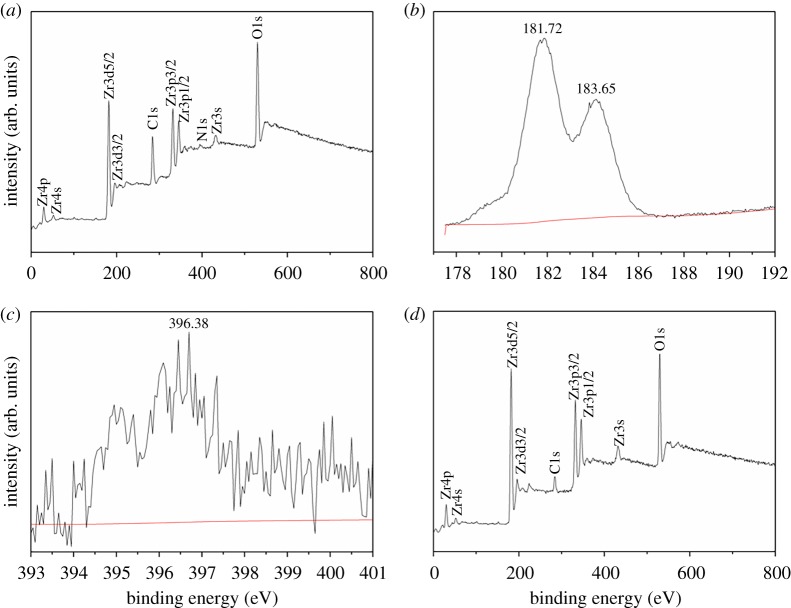
XPS analyses of the as-prepared ZrN sample and reaction product of ZrN and NO at 600°C. (*a*) Survey spectrum of ZrN, (*b*) Zr3d region of ZrN, (*c*) N1 s region of ZrN and (*d*) survey spectrum of the reaction product after the reaction of ZrN and NO at 600°C.

The as-prepared ZrN sample was characterized by XPS measurements. From the survey spectrum (figure 1*a*), one can readily identify Zr 3s electrons at 421.36 eV, Zr 3d electrons at 181.72 and 183.65 eV, Zr 4s electrons at 58.31 eV, Zr 4p electrons at 33.15 eV and N 1s electrons at 396.38 eV (the O 1s electrons at 529.15 eV suggests surface partial oxidation, whereas the C 1s electron at 179.25 eV likely arose from residual carbon). In fact, high-resolution scans show a doublet at 181.72 and 183.65 eV for the Zr 3d electrons (figure 1*b*) and N 1s electrons at 396.38 eV (figure 1*c*), consistent with those of ZrN [32]. Furthermore, based on the integrated peak areas, the mole ratio of Zr/N is estimated to be 1 : 0.87, which is close to that of ZrN.

### 3.2. Powder X-ray diffraction characterization

**Figure 2.**
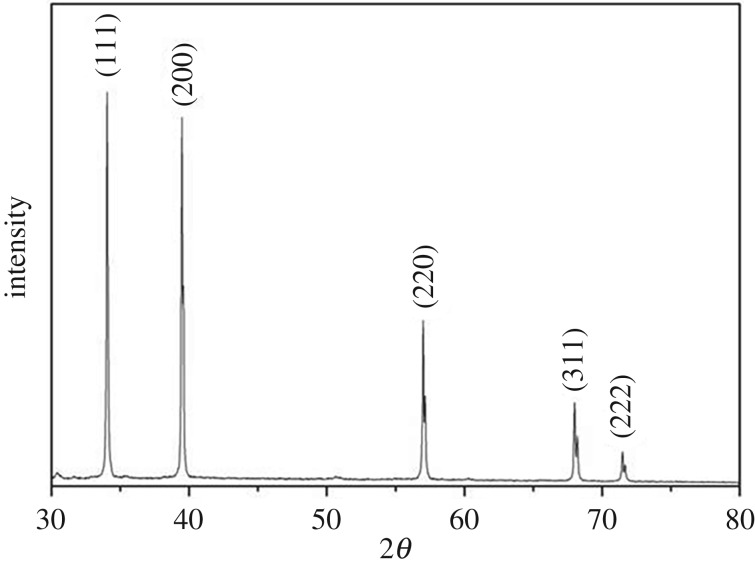
XRD pattern of the as-prepared ZrN sample.

XRD measurements of the ZrN sample (figure 2) show five well-defined diffraction peaks, which correspond to a d-spacing of 2.6310, 2.2799, 1.6145, 1.3779 and 1.3191 Å, and can be indexed to the (111), (200), (220), (311) and (222) crystalline planes of cubic phase ZrN (space group Fm3 m, no. 225). The lattice constant of *a* = 4.5675 Å is in good agreement with the JCPDS card no. 35-0753 [33–34]. This suggests that the produced ZrN powders possessed good crystallinity.

### 3.3. Transmission electron microscopy characterization

**Figure 3.**
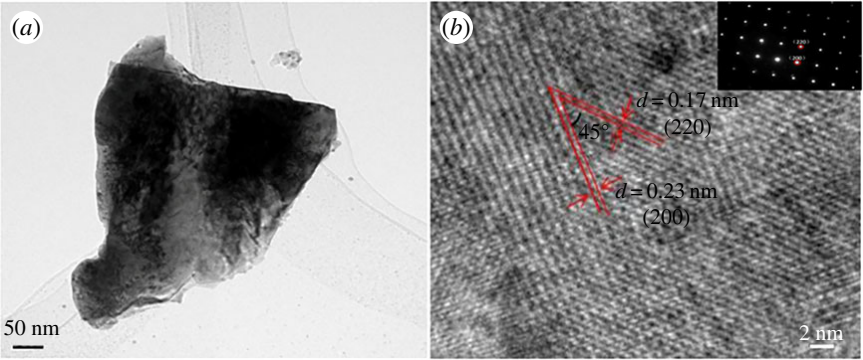
(*a*) TEM and (*b*) HRTEM images of the as-prepared ZrN. Inset to panel (*b*) is the corresponding SAED pattern.

To elucidate the microscopic structure of the ZrN sample, we conducted a detailed TEM investigation. Figure 3 depicts two representative TEM micrographs at different magnifications. From figure 3*a*, it can be found that the morphology of the ZrN was of irregular particles with a diameter of 250–300 nm and a thickness of 20–30 nm. The corresponding HRTEM image is shown in figure 3*b*, which exhibited well-defined lattice fringes with two interplanar distances of 0.17 and 0.23 nm, corresponding to the (220) and (200) crystalline planes in cubic ZrN at a crossing angle of approximately 45°. The corresponding SAED pattern (inset to figure 3*b*) is also in good agreement with crystalline cubic ZrN [35].

### 3.4. Elimination reduction of nitric oxide

**Figure 4.**
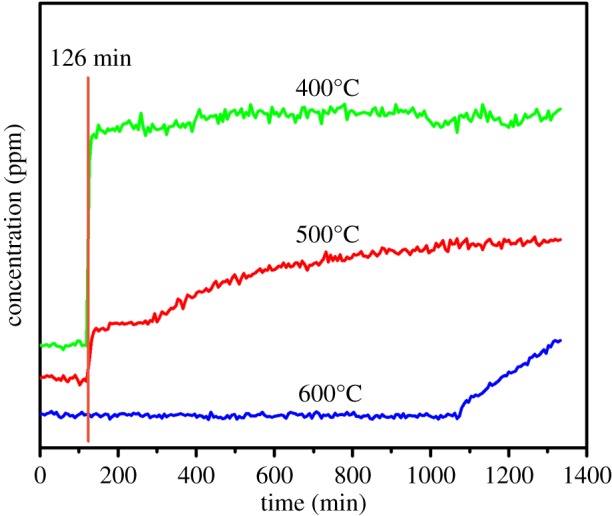
Degree of NO removal by ZrN at different reaction temperatures.

Figure 4 depicts the removal of NO by ZrN at different reaction temperatures. Experimentally, a gas containing 500 ppm NO balanced with N_2_ was introduced into the reactor after 126 min, and the reaction was performed at different temperatures. One can see that with the increase of reaction temperature from 400 to 600°C, the efficiency of NO conversion over ZrN increased markedly (figure 4). Note that at 400°C, the concentration of NO at the outlet was equal to that at the inlet, suggesting no reactivity at this temperature. When the reaction temperature was increased to 500°C, the amount of NO detected at the outlet decreased slightly, indicating partial reduction of NO by ZrN. When the temperature was further raised to 600°C, no NO was detected at the outlet, indicating that NO was completely reduced by ZrN in the reactor. The conversion rate of NO in this reaction is much better than those reported with other catalysts such as CuO*_x_*/Al_2_O_3_ and CuO*_x_*/LaO*_x_*/Al_2_O_3_ [11], suggesting that ZrN can be used in gasoline vehicle exhaust treatment at high temperatures.

**Figure 5.**
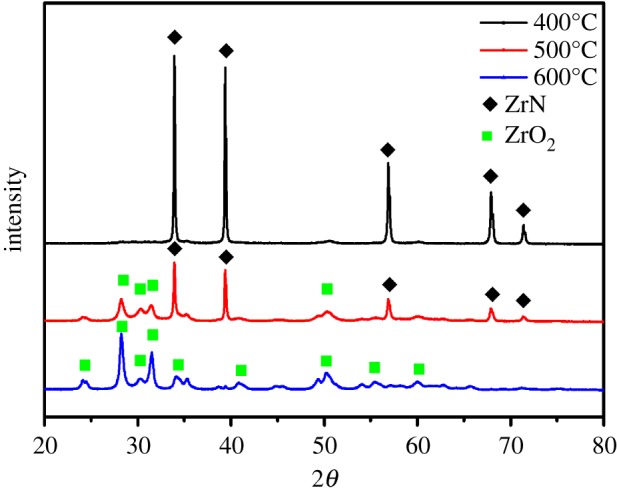
XRD patterns of the solids after NO reduction reaction at different temperatures.

Consistent results were obtained in XRD measurements of the solids before and after NO reaction (figure 5). When the reaction was carried out at 400°C, no variation of the diffraction patterns of the solids was observed, in agreement with the lack of chemical reactivity at this temperature. When the reaction temperature was raised to 500°C, the solids were found to consist of a mixture of ZrN and ZrO_2_, indicating that part of the ZrN was oxidized into ZrO_2_ during the reduction of NO to N_2_. At the even higher reaction temperature of 600°C, no ZrN was detected and only ZrO_2_ diffraction peaks were observed. Consistent results were obtained in XPS measurements of the solids after reaction (figure 1*d*), due to complete consumption of ZrN in the reduction of NO: 2ZrN + 4NO = 2ZrO_2_ + 3N_2_.

**Figure 6.**
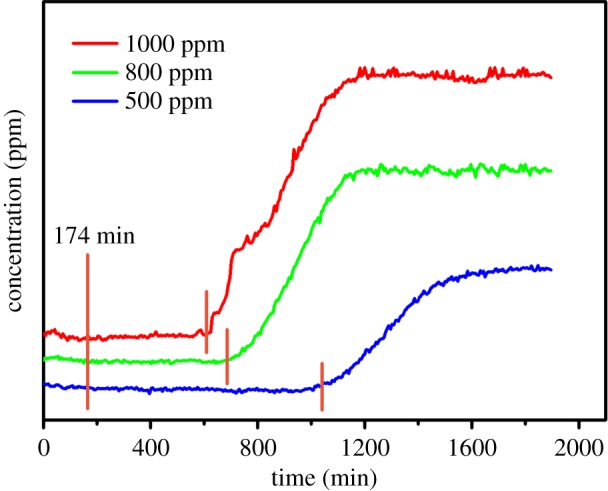
Reaction curves of ZrN with different concentrations of NO at 600°C.

In further studies, reduction of NO by ZrN was carried out at 600°C with different concentrations of NO gas (500, 800 and 1000 ppm), where the gas was introduced into the reactor after 174 min, as depicted in figure 6. We can clearly see that at the gas concentration of 500 ppm, the amount of the added ZrN was sufficient to completely reduce NO for about 14 h. At increasing NO concentrations, the period of time where complete reduction of NO occurred was shortened accordingly. For instance, when the gas concentration increased to 800 and 1000 ppm, the operation time was shortened to 8.4 and 7.5 h, respectively.

## 4. Conclusion

In summary, ZrN powders were prepared by thermal treatment of Zr in a N_2_ atmosphere at elevated temperatures, and exhibited apparent activity in the reduction of NO to N_2_ where ZrN was oxidized to ZrO_2_. The experimental results show that 0.5 g of ZrN powders was sufficient for complete reduction of 500 ppm NO gas for 14 h at 600°C, whereas the chemical reactivity diminished with decreasing temperature. The reaction products were detected as ZrO_2_ and N_2_, both of which are of practical value and cause no secondary pollution to the environment. Importantly, because of the high conversion rate of NO at elevated operation temperature, ZrN may be used as an effective substitute of conventional catalysts in the treatment of gasoline vehicle exhaust gas. These results highlight a new, unique approach based on the concept of green chemistry for the effective elimination of NO*_x_* pollutants.

## Data accessibility

All data generated or analysed during this study are included in this article.
